# Deciding Together? Best Interests and Shared Decision-Making in Paediatric Intensive Care

**DOI:** 10.1007/s10728-013-0267-y

**Published:** 2013-11-22

**Authors:** Giles Birchley

**Affiliations:** Centre for Ethics in Medicine, University of Bristol, Canynge Hall, 39 Whatley Road, Bristol, BS8 2PS UK

**Keywords:** Best interests, Children, Compromise, Ethics, Intensive care, Shared decision making

## Abstract

In the western healthcare, shared decision making has become the orthodox approach to making healthcare choices as a way of promoting patient autonomy. Despite the fact that the autonomy paradigm is poorly suited to paediatric decision making, such an approach is enshrined in English common law. When reaching moral decisions, for instance when it is unclear whether treatment or non-treatment will serve a child’s best interests, shared decision making is particularly questionable because agreement does not ensure moral validity. With reference to current common law and focusing on intensive care practice, this paper investigates what claims shared decision making may have to legitimacy in a paediatric intensive care setting. Drawing on key texts, I suggest these identify advantages to parents and clinicians but not to the child who is the subject of the decision. Without evidence that shared decision making increases the quality of the decision that is being made, it appears that a focus on the shared nature of a decision does not cohere with the principle that the best interests of the child should remain paramount. In the face of significant pressures toward the displacement of the child’s interests in a shared decision, advantages of a shared decision to decisional quality require elucidation. Although a number of arguments of this nature may have potential, should no such advantages be demonstrable we have cause to revise our commitment to either shared decision making or the paramountcy of the child in these circumstances.

## Introduction

Shared decision-making (SDM) is widely advocated within current healthcare practice, but its legitimacy as a model of decision-making for infants and other incompetent patients has been questioned [76]. Despite this, when making decisions for children, English law^1^ says clinicians and parents should try to agree where the child’s best interests lie. Moral legitimacy does not necessarily depend on the agreement of interested parties, so just agreeing a child’s best interests does not guarantee that they are really being served. While this problem might be addressed by including the child within the decision making process,^2^ in environments such as paediatric intensive care where most patients are either too young or too disabled to take part this is impossible. Because of this, where the child has no antecedent wishes and is either too young or too disabled to otherwise take part, the moral legitimacy of SDM between parents and clinicians in the determination of a child’s best interests (what I term *paediatric shared decision*-*making*—PSDM) requires consideration. Although scholars debate the degree to which the best interests^3^ test is objective or philosophically robust [5, 10, 37], the moral legitimacy of the model of PSDM promoted in English law has yet to be considered. This paper argues that PSDM may be incoherent with the claim that the interests of children are paramount, a central claim in legal conceptions of best interests.^4^

Focusing on disagreements about a child’s quality of life where it is disputed whether intensive care or withdrawal serves a child’s best interests, I detail how current English law suggests resolving such disputes with PSDM. While SDM has been widely promoted within healthcare practice, the impetus for this has been to assure the autonomy of patients is respected in the clinical encounter. Yet in PSDM the autonomy rationale is incoherent because the child has no known wishes or feelings on which parent proxies may base their authority. Instead, PSDM appears more roundly to be justified by the contentious nature of unilateral decision-making, since the locus of moral authority does not appear to lie clearly with any decision-making party. Apparent advantages for active participants of PSDM, as well as society at large, are contained in the literature, yet there are no suggestions that the child might benefit by, say, a better decision being made. Indeed, since agreement, rather than the quality of the decision, is the focus of PSDM, it seems incongruent with the claim that the best interests of the child are paramount, especially since the opacity of children’s best interests means there are already risks these will be conflated with one or other negotiating party. Further, since the literature also speculates that SDM imposes demanding criteria upon decision makers, but evidence shows the courts are rarely approached, it is unclear if decision makers are able to collaborate effectively in practice, or if this further undermines the interests of children. Although I sketch reasons why there may be advantages for the child in PSDM, they require deeper elucidation if we are to robustly defend this approach; absent such reasons, if we remain committed to PSDM, we must re-evaluate the way we promote the child’s best interests.

## Best Interests in English Law

The best interests test has been in English statute for almost a century^5^ and is a widespread international standard as a result of the near unanimous adoption of the United Nations Convention on the Rights of the Child [84]. In paediatric healthcare English law favours a process in which parents and clinicians agree a common understanding of the child’s best interests. If agreement is impossible, a judge determines the “objective” interests of the child and rules appropriately.^6^

Medical law reaches this standpoint through several cases in which children’s best interests were seen to extend beyond medical interests. *Re J* [66] has become the leading case on the matter: J was born at 27 weeks gestation and his prematurity caused brain damage, convulsions and spastic quadriplegia. His doctors wished to withhold artificial ventilation but were opposed by the Official Solicitor. The Court of Appeal ruled that because J had an extremely poor quality of life and no prospect of improvement, withholding ventilation was in his best interests. *Re J* established that the parents’ opinions of a child’s best interests should be considered as part of a joint decision:

No one can dictate the treatment to be given to the child, neither court, parents nor doctors. There are checks and balances. The doctors can recommend treatment A in preference to treatment B. They can also refuse to adopt treatment C on the grounds that it is medically contra-indicated or for some other reason is a treatment which they could not conscientiously administer. The court or parents for their part can refuse to consent to treatment A or B or both, but cannot insist on treatment C. The inevitable and desirable result is that choice of treatment is in some measure a joint decision of the doctors and the court or parents^7^

Subsequent developments in adult jurisprudence,^8^ caused best interests to be further clarified in *Wyatt* [59]^9^ to include issues of emotion and welfare as well as medical considerations, and by the time of *Glass v UK* [30] a collaborative approach was already usual in practice.^10^ *Glass v UK* concerned 15 years old David Glass, who had spastic quadriplegia, hydrocephalus and severe learning difficulties, and had been recurrently admitted to intensive care with chest infections. Believing David was in the terminal phase of recurrent respiratory illness, doctors began a diamorphine infusion despite his family’s opposition, refused to let them take David home to die and, without seeking their consent, placed a “do not resuscitate” order in his medical notes. The European Court of Human Rights ruled that, in neither seeking a proxy’s consent for his treatment, nor approaching the courts for an appropriate order, David’s article 8 rights to privacy and family life had been violated. The effect was to underline both the authoritative nature parental consent to treatment, and the ultimate authority of the courts where this parental authority was to be challenged.

Although medical views of best interests generally prevail, the case of *MB* [2] was a clear demonstration of the courts’ ability to substitute its own opinion on these matters.^11^ In this case, doctors sought to fatally withdraw ventilation from an infant with spinal muscular atrophy, a degenerative disease that causes progressive muscle weakness, leading to ventilator dependency and, eventually, complete paralysis. Refusing to grant legal permission for withdrawal, Holman LJ suggested MB’s relationship with his family and the pleasures he derived from sensory stimulation were not outweighed by the pain and discomfort of his ongoing care. Instead he proposed a compromise, in which MB’s ventilation and everyday care continued, but further aggressive measures that would keep him alive were withheld.^12^

*Re J*, *Glass v UK*, and *MB* are of interest, because they involve judgement of best interests where there is apparently legitimate ethical dispute about whether the child’s life is worth living if treatment is continued, rather than whether medical treatment would be effective.^13^ The evolving judicial approach in these cases, particularly that seen in *MB*, may be to arrive at some form of compromise where each party’s point of view is taken into account in the final decision. I will consider if this legal process is ethically supportable, yet there remains a further question of how it manifests in practice. This is difficult to determine, because, while court judgments are within the public domain, it is apparent the vast majority of cases reach a solution through parent-clinician negotiation. Brierley et al. [11] provide a rare insight into the negotiation process within one large paediatric intensive care unit. Over a 3 years period, in all but seventeen of 203 cases where clinicians recommended withdrawal of life sustaining treatment, parents agreed. Of the seventeen disputed cases, only one reached the courts, the remainder being resolved either through unusually protracted negotiation or the death of the child because of their illness. This picture is born out in the court records where only six similar disputes appear to have been reported since *MB* [2] in 2006.^14^ While, if we take avoidance of the courts as a mark of success, these figures suggest parties may be reaching amicable agreement, without empirical investigation it is impossible to conclude whether it is on an ethically defensible basis. Certainly a large number of factors might potentially favour intransigence on the part of one or other decision maker in the case of conflict. Pressures such as the cost and scarcity of an inpatient bed and the evolving nature of illness may play a large role in such negotiation, and an obdurate party may gain undue influence over the process.^15^

In order to decide if PSDM is morally legitimate, we need empirical investigation of the content of bedside decisions.^16^ We must also answer some normative questions. If we are to defend PSDM we must suggest why decisions arrived at from intransigence are less morally defensible than those that were arrived at by PSDM, for, presuming the decision is right, why should coercion be morally reprehensible, if the child’s interests are paramount? The answers to these questions lie in considering the uncertain locus of moral authority, the clarity with which we can identify children’s interests, and the urgency of the situation. Before we turn to these concerns, let us briefly clarify the concept of SDM within healthcare generally and consider its awkward relationship to paediatrics.

## The Concept of Shared Decision-Making

Over several decades, SDM has become an increasingly important feature in healthcare as a way of ensuring patient autonomy, although it has been subject to a degree of conceptual confusion [15, 26, 51].^17^ In a widely cited typology of decision-making, Charles et al. [15] identify unilateral models where the locus of control is either with the clinician or the patient. In paternalism, the doctor makes the decision they think best. In the informed patient model, the doctor is relegated to information giver, and the patient makes the decision. In the professional-as-agent model, the doctor, aware of the patient’s health needs^18^ and furnished with the patient’s social situation and values, makes the decision that serves them all. Charles et al. [15] differentiate SDM from these unilateral models by its involvement of two or more willing participants who share information to reach a consensual decision. This description raises an immediate problem when the decision is focused upon an infant, since SDM describes this process only if we are willing for another to participate on the patient’s behalf. Thus, in an extensive investigation of the ethics of SDM, Munthe et al. [51] observe that although the absence of autonomy in children raises complications in SDM, these are generally solved using models of family and relationship-centred care. I argue presently these do not ensure the needs of the child are paramount. While the authors acknowledge that there may be increased conflicts of interest when we involve those with external interests in the patient’s wellbeing they do not concentrate their argument on this point. While the autonomy model is apparently poorly calibrated to paediatrics, the same paper lists a number of other benefits of SDM, including the strengthening of clinical partnerships. These are admittedly important to paediatrics because lack of parental compliance may present challenges to effective treatment of the child and thus maintenance of trust between clinicians and parents is a concern. There are also significant drawbacks in unilateral decision-making that are specific to making decisions for infants. These rest on the uncertain locus of moral authority and represent strong reasons to collaborate.

## Drawbacks of Unilateral Decisions in Paediatric Healthcare

The location of moral authority in paediatric decision-making is a central point of contention in the bioethical critique of best interests which encompasses a critique of unilateral decision-making models akin to the typology presented by Charles et al. [15]. The paternalistic standpoint argues that clinicians are best placed to make a decision on the child’s behalf since they have the clearest understanding of a child’s medical interests, and can glean other relevant information about the values of the family, albeit such information is subsidiary, since the child is the focus of the decision. Akin to the informed patient model is familialism. Because families are naturally beneficent to their children and their interests closely allied, given the necessary medical knowledge parents will have the clearest understanding of their child’s overall interests, so should unilaterally make any decision. We can add to these models what I term the ‘judicial approach’, which suggests that a neutral arbiter, usually a judge, should make a final decision, rather than a doctor or a family. Each of these unilateral approaches involves the communication of all the pieces of the informational jigsaw to a single party, who then acts as arbiter to produce a final decision.^19^ Such approaches may seem an attractive way to remedy dispute, as they avoid the difficulties inherent in PSDM. However, since neither clinicians, parents nor judges have clear moral authority in a best interests decision with a strong moral component, such as those exemplified by the *Re J* [66], *Glass* [30] and *MB* [2] cases, unilateral approaches present problems of moral validity.^20^

While the careful balance of evidence and rational argument in common law suggests it contains elements we would expect to find in moral reasoning, legality has an ambivalent relation to moral standing that suggests the judicial approach may fail the test of moral validity. The dominant theoretical underpinning of positive law suggests that there should be a separation of law and morality, and, although there is arguably a moral component in many judgments, judges may disavow this.^21^ Certainly, working within the principle of *stare decisis* a court must conform with precedent in a process that has little to do with moral reasoning. Paternalism is also challengeable since medical interests are not all that are relevant to a child’s best interests (there are types of good life involving sub-optimal health) and the expertise of clinicians in medicine does not confer expertise in morality [9]. Meanwhile, although a broadly based familialism has many advocates in bioethics [25, 39, 41, 71], the key assumption that intimate families are motivated by beneficence [71] is contestable both because such beneficence is inherently controversial (since we cannot access what is in the best interests of the child) and also because of the motivation of families who abuse their children may be maleficent [44]. Accepting this, some suggest adopting a threshold of family autonomy, below which positive steps to intervene may be made. A wide variety of such thresholds have been proposed including reasonableness [25], basic interests [47, 71], uncertainty [39] and harm [21, 22, 25], yet these measures are not necessarily clear. The divided academic response to legal notions of reasonableness is testament to its contestability,^22^ and uncertainty and basic interests are not self evidently defensible measures.^23^ A harm threshold alone may guard children from neglect^24^ but is a contentious way to limit intensive care since death represents a harm of indeterminable value.

Similarly, the partialities of each decision-making group may cause their judgement to be questioned. Judges have been criticised for their lack of diversity, conservative social attitudes [5, 8] and deference to medicine [87]. Clinicians appear more pessimistic about the quality of life of disabled children than either parents or the children themselves [80]. Practitioners may feel profound grief on the death of a child [58], and it has been argued that repeated exposure to such emotions may lead practitioners to aim for outcomes, such as treatment withdrawal, where they feel they ultimately limit their regret, rather than risk emotional turmoil by taking a chance on a favourable outcome that does not materialise [83]. Few families wish to harm their children, but the instinctive desire to protect a child’s life may prevent families from rational consideration of the seriousness of an illness or the hopelessness of a situation [82] and lead to overestimation of their own capacities as long term carers [88]. There may also be considerable resource implications in empowering parents over clinicians that affect wider health provision [51].

A further problem is that it is not self evident if treatment or non-treatment serves best interests, since personal values dictate the relative desirability of death and life. Many authors note an explicit link between best interests and the quality of life [14, 40, 81, 85] a link that manifests because we must apparently judge a child’s interests on some measurable basis. Such criterion feature in practice guidelines [56, 72], and are widely considered in practice [85]. Doubts remain over the validity of such measures; self-reported quality of life in both children with disabilities [57, 73] and families of very low birth weight children [79, 80] suggests these individuals’ quality of life is no different from their peers, particularly when adequate support is in place. Although we must be cautious about generalising these data inappropriately [34], it must give us pause to reconsider negative intuitions where quality of life is in question,^25^ as well as to reflect that the lack of longitudinal tracking of controversial outcomes is a severe impediment to our ability to learn from experience.^26^

To these uncertainties we can add the need to make a decision urgently to avoid negative consequences for both the patient and wider society. Society suffers because resources will be expended. The patient suffers because the discomfort associated with intensive care is considerable. Ventilation and intravenous lines are encumbering, painful and distressing, despite routine medication for pain and anxiety, the use of which is restricted by problems with tolerance and withdrawal [55]. Such traumas can cause psychosis and long term psychological disturbance in intensive care survivors [18] and clinicians are quick to describe futile intensive care as torture and an affront to human dignity [11]. If we accept the child has interests, we must also accept that doing nothing will affect them.

These issues of moral authority, non-self evident interests and urgency represent extensive reasons against either unilateral decision-making or deferring the decision. Yet these are negative reasons, rather than representing the positive benefits of SDM. There are prudential reasons for including all parties; for instance, the shared stake in maintaining civil society that lies behind the perpetuation of the rule of law may serve to defend a judicial approach. A desire to maintain the institution of the family as a core, self supporting social unit may encourage familialism. A belief in the moral mission of medicine may suggest medical paternalism. The current legal tendency toward PSDM may represent a marriage of these interests for the sake of a harmonious society. But this prudential basis seems unsatisfactory if the needs of the child are to be paramount. While it could be argued that it is for the individual collaborators to prioritise these needs, a purely prudential explanation of PSDM seems incongruent with the paramountcy principle because it focuses only upon the needs of collaborators rather than the quality of the decision.^27^ This may be a particular problem since the best interests test already seems to be afflicted by a special opacity. By claiming that the interests of children are of special importance, it has been argued that the best interests test precludes expression of any other legitimate interests [12], encouraging dishonest expression of all interests as being in terms of the child [83]. Furthermore, as best interests only allow the expression of a single set of interests that are arrived at in an opaque manner, these interests may be hijacked by another party who claims their interests concur with the child’s [16]. Of course, we may argue that PSDM is neither concerned with nor responsible for these problems, and it is legitimate for a process to consider the needs of the negotiating parties first and foremost. Yet the child’s interests may be very slender and they are voiceless and may be easily forgotten. Given the significant pressures towards conflating outside interests with those of the child, it would be more satisfactory (and certainly more congruent with paramountcy) if we could demonstrate that PSDM had positive benefits for the child. The existence of such positive benefits does not seem so farfetched; let us explore whether the literature on SDM can contribute such reasons.

## Models of Shared Decision-Making

Munthe and Sandman [51, 75, 76] have published extensively on the subject of SDM. In a typology of SDM models [75], these authors detail among others the *shared rational deliberative joint decision model* (SJDM) and the *professionally driven best interests compromise model* (PBCM). In the former model, a free ranging discourse between the doctor and patient is envisaged, which results in a consensus decision. This discourse is characterised by complete equality and openness between the parties up to and including allowing their own interests to be challenged and perhaps discarded in favour of the other. In contrast, the PBCM pragmatically dispenses with equality and allows the doctor to take strategic action to control the negotiation, so long as the doctor’s goal is to advance the patient’s best interests. This is done by giving the patient a frame of choices, modulated by wider healthcare values, that range from that which is in their medical best interests to that which merely satisfies a minimum medical standard. Since paternalism is inefficient in advancing such interests (for instance by undermining the therapeutic partnership) and does not respect autonomy, the strategic action that PBCM allows the doctor must be openly communicated to the patient. While suggesting that SJDM represents the ideal model, In the face of the considerable demands it makes of participants, Sandman and Munthe [75] argue that PBCM represents a more achievable balance between the need to guard both the best interests and the autonomy of the patient.

PBCM is appealing in PSDM, since it seems to satisfy a parent’s need for control while allowing the clinician to safeguard the child’s interests, perhaps by offering a choice between two clinical therapies, or by setting limits when a treatment is definitely painful, distressing or medically inappropriate. Yet it raises concerns as well. The least of these is that we will satisfice, rather than maximise, the child’s interests. Although diverging from the letter of the law, many bioethicists have argued that satisficing is the de facto purpose of the best interests test [13, 42]. More importantly PBCM seems to offer a reductionist account of what is at stake in healthcare disputes. Healthcare disputes rarely involve a bald choice between the effectiveness of therapy A and therapy B, with all other things remaining equal. The human condition is complex and frequently there are also moral elements at play. In such circumstances PBCM seems vulnerable to the same criticism as paternalism, because clinicians are not moral experts. Sufficiently differentiating PBCM from paternalism is difficult in these cases, even when allowing some flexibility. For instance it has been noted that medical views of treatment limits may significantly differ from families’ judgements of when pain, distress or appropriateness outweigh the value of life or the chance of recovery [86]. These differences may be so great that the range of clinically acceptable options may completely fail to coincide with what the family finds acceptable, leaving only the judicial approach to dispute resolution effective.^28^ Accepting this, we return to SJDM, where the aim is a free consensus between the negotiating parties. The authors list a number of benefits for the patient, including improved wellbeing, psychological benefits and improved compliance [51],^29^ yet we must ask if these benefits apply to the non-autonomous infant or to their parent. Granted, such a separation is often difficult to make since benefits to the parent may benefit the child: parental wellbeing should improve their ability to care for the child and to interact with clinicians. A child whose parents are non-compliant is more likely to themselves be non-compliant. Yet the risk here is that we take this conflated benefit for granted. There is clearly a point at which parental compliance and wellbeing may be bought at a cost to the child. Furthermore, a parent who uses their influence upon the child as leverage in a negotiation is clearly not partaking in SJDM but in a process of bargaining that suggests a certain amount of bad faith. There may be further benefits to SJDM. As a consensus model, it is similar to models of *moral* SDM, which focus upon equitable ways to resolve the moral, rather than the clinical, aspects of disputes. These also offer valuable reflections upon SDM at large.

## Moral Shared Decision-Making

Moral SDM encompasses models that characterise SDM as negotiation.^30^ Huxtable [38] suggests we might solve moral dilemmas using *moral compromise* where parties trade concessions in order to reach a shared decision that advances some of their original interests. Moreno [48] provides a contrasting model, *moral consensus*. This requires the negotiants to enter the process of moral SDM free of a fixed agenda and open to entirely new perspectives in a way conceptually similar to SJDM. Moreno suggests consensus is distinguished from compromise by its untethering of a solution from the fixed interests of participants. However, Moreno readily acknowledges that some moral disputes are too entrenched to meet these demands,^31^ and the best that can be hoped for is a compromise position; indeed, these criticisms notwithstanding, compromise and consensus are readily conflated, and at times Moreno’s own careful attempts to distinguish them appear to fail.^32^ Furthermore, Huxtable also envisages some abandonment of fixed starting positions, so for the purposes of the ongoing discussion I suggest that the analysis on which both compromise and consensus is based is sufficiently similar for the distinction not to concern us. I shall base my discussion on Huxtable’s analysis, which offers a typology of reasons for compromise.^33^

Compromise and consensus have each been criticised as flawed and anathema to moral debate. While consensus has been criticised for stifling dissent, Moreno counters that, without some eventual point of agreement, dissent is self defeating [48]. Holm [36] suggests that compromise either muddles our understanding of what is good or represents an unacceptable surrender of our core moral beliefs. Taking Moreno’s argument further, Huxtable [38] counters with reasons compromise is morally superior to endless dissent. First, in compromise we prudentially advance some of our aims. Second, where we lack resources to honour all claims, we can only justly share by compromise. Third, some decisions cannot defensibly be abandoned. Fourth, it serves the necessity of co-existence between disputants. Fifth, the uncertain defensibility of ethical decisions mean we should consider other points of view based on alternatives. Lastly, the complexity of moral decisions means we may fail to see potential deficiencies of our own reasoning, and potential strengths in others’.

Because compromise entails collaborators abandoning their moral starting positions to reach an agreement for potentially prudential reasons, Huxtable proposes criteria to mark morally defensible compromise. Echoing suggestions in SJDM and consensus, to prevent compromise being used as a combative process to extract the maximum from a situation, participants must be reflective, their agenda must be transparent and they must be respectful of the moral worth of the other party. Since all these models suggest fairly demanding conditions for collaborators, let us first consider the practical problems these demands pose, before we examine what compromise offers a defence of SDM.

## Risks and Benefits

Shared decision-making (SDM) is not incontestably beneficial, and Munthe and Sandman have done much to elucidate the risks it may involve, as well as questioning the presumption that patients may be able or willing to actualise their autonomy [51]. Similarly moral SDM is a demanding process that entails equality and abandonment of personal interests, [48, 75] as well as reflexivity, transparency and respect for the other party [38]. Although a presumption against these abilities seems premature given the lack of empirical information about PSDM at the bedside, it nevertheless seems likely that clinicians and parents will not always fulfil these criteria. Impossibly demanding criteria may leave unchecked outside pressures toward displacing children’s interests and the hijacking of children’s interests by others. While we must acknowledge potential goods in PSDM, we must also carefully weight these against the risks involved, and either take steps to reform PSDM to avoid them or to consider if children’s interests should be inviolable. We must also make these processes as effective as possible, and consider the ability of parents and clinicians to satisfy demanding criteria alone. The fact that such demands seem more easily attainable by disinterested parties leads Huxtable to focus his arguments upon SDM that has entered the courtroom, and it is worthwhile to consider whether a similar venue is necessary for effective SDM. Moreno alerts us to the work of the sociologist Georg Simmel [78], who considers the attributes of groups. Simmel suggests that groups of three (triads) possess unique qualities of intimacy, mediation and durability that are lacking in groups of two (dyads). This observation may suggest that PSDM involving a dyad of parents and clinicians will be more vulnerable to breakdown in negotiation and the addition of a third party may create a beneficial dynamic. The identity of this party is moot; however, one may speculate that this party would need to be both mutually respected and independent. Since these qualities are found in the judicial process, perhaps it should be involved more routinely.^34^

While the demanding nature of these models represents a practical problem, moral SDM may offer strong reasons against unilateral decisions. These reasons are drawn of either necessity, or a desire for a harmonious, equitable society—prominently seen in the suggestion that we can achieve some of our aims, and negotiate our share of scarce resources. Yet again these only count as negative reasons against unilateral decisions, rather than positive reasons for SDM. If we search for such reasons, the overarching argument is that moral SDM conforms to the pluralistic tenets of a liberal society. These suggest that the moral foundations of a democratic society must be based on respect for individuals, equitability and peaceful settlement of disputes; in other words, a vision of a moral society that is predicated upon a vision of societal self interest.^35^ Moral liberals may therefore claim that a society that values SDM is just and respects autonomy; this benefits all parties who share the decision and belong to such a society. However this explanation lacks reasons why moral SDM will produce morally valid answers when we are making decisions on behalf of persons with unknown wishes. This focus on the process of decision making rather than the quality of the decision may be due to deficiencies in the model of pluralism upon which such theories are built [7, 38, 48]. This model tends to be Rawls’ *reasonable pluralism* [61], which suggests that because people have different goals, psychologies and experiences, it will be impossible even for those who are honest, reasonable and try their best to be unbiased to agree about their responses to every moral situation. Because of this disagreement, if we wish to avoid coercion we must focus on areas of consensus and accept that areas outside this consensus will remain the subject of dispute. Rawls avoids a collapse into relativism, suggesting that although consensus on many moral principles is not required, we must triangulate our principles with a just political regime that treats individuals fairly. Although I shall not dispute the coherence of this theory in consideration of moral agents who are capable of expressing their wishes, it appears deficient when used to justify making decisions on behalf of those whose wishes are unknown. I have already argued that, as individuals, doctors, parents and judges lack the moral authority to be sole arbiters of moral disputes. What we still lack is a robust explanation of why PSDM collectively imbues them with this authority, and pluralism is deficient on this count.^36^

## Qualities of Shared Decisions

The theories presented then, fail to expound ways in which PSDM may offer a better quality of decision to the infant who is its subject. These problems may not be insoluble although I suggest that further philosophical research is needed to explain why this might be so in moral disputes. This is no mean undertaking since it involves an exploration of the nature of epistemological and moral reality that is beyond the scope of this paper. Nevertheless, one might sketch some of the features of such an explanation. For instance, it is often taken for granted that widespread agreement enhances accuracy, and certainly statistical agreement of independent physical measurements is more likely to indicate correctness than error. Yet how such observations affect the verification of ethical viewpoints reached by SDM requires subscription to certain epistemological and philosophical concepts. One argument may embrace Nagel’s [52] gradualist paradigm of objectivity, which suggests that we may consider positions more or less objective if they have some of the features we expect of objectivity [50]. If we then accept that moral facts are real and discoverable, and that rationality is the key to this discovery, we can claim the more independent rational arguments that agree on a particular position the closer we have come to an objective reality. Another argument suggests that group decisions may reduce individual bias, especially if the opinions within the group are diverse. Evidence for the psychological basis of this has been discussed elsewhere [32], although we must balance this by noting that we have long known that individuals are susceptible to group pressures to conform, especially if they are a lone voice [49]. A further argument may be that a more complex appreciation of the consequences of a best interest decision may be elucidated if we consult more widely. However, depending on our understanding of ethics, consequences may vary in importance.^37^ These arguments may be extended, but none are yet completely compelling, even if we accept the philosophical commitments they require. For the present, perhaps the best we can say at present is that, in combination with other methods such as empirical fact finding, shared decisions may sometimes be better than those taken unilaterally.^38^

## Scope for Reform

Whatever the approach taken, making decisions in the best interests of children presents a number of challenges. In cases where the decision concerns whether arduous and prolonged treatment is for the best, and where the chances of survival are marginal and it is expected that the child will have a severe impairment should they recover, these challenges are even more acute. Such decisions depend not only on medical prognostication and the chances of recovery (and what a ‘good’ chance is may not be the same to both doctors and parents), but how much value there is in a life of acute disability. Where the wishes of the child are inaccessible, we have no recourse to the principle of autonomy to help us. Naturally, parents and doctors will have their own opinions, and the fact that parents are closely bonded to the child and are irrevocably affected by the outcome means that it seems reasonable to include their opinions. In these circumstances, the principle of paramountcy, even in its mildest form, suggests that the decision making process must value children and ensures their voices are not marginalised. In the legal context Choudhry and Fenwick [17] have argued that the basic tenets of the paramountcy principle, by favouring the child above all others, sharply contradicts the presumptive equality of competing rights that is enshrined in the Human Rights Act, suggesting paramountcy is ripe for reform. Yet defending the principle Herring [35] suggests this it remains important because children are often unable to promote their own interests, and without some formal priority, children’s interests are easily overlooked. The basic voicelessness of children is a strong argument that a reasonable criteria for PSDM should sacrifice neither the benefits to participants of SDM, nor the safeguards to the child expressed within the principle of paramountcy.

It is nevertheless easy to offer arguments which support abandoning a formal commitment to paramountcy For instance we may argue that although PSDM does not offer a superior moral position, neither is it inferior, and that the benefits it offers negotiating parties are significant enough to support its use. Although it seems philosophically correct to consider that the child has separate and identifiable interests, these are inaccessible and paramountcy is rhetoric and pretence. On the other hand, we may respond that the best interests principle is not a practical position but a guiding ideal to promote the interests of children [35, 42]. From this point of view, the problem with PSDM is that implementation of this ideal is too opaque, and a more transparent method should be considered. Although the gap between children’s welfare and rights is not always easily defined, some have suggested that open recognition that the interests of children and parents may conflict encourages a transparent approach to making decisions on behalf of children. A rights based approach does not preclude expression of the competing values at work, allowing all claims to be considered fairly [6, 23, 24] and the rights of the child to be aired and appropriately weighted. While the question of who should determine the child’s rights is not without its own problems [35], such an approach would give the child an independent voice in PSDM.

## Conclusion

Understood broadly, the best interests test means that the interests of children should never be forgotten within a world of adult concerns [35]. Although it is questionable that SDM can be instrumental in promoting these interests, the English courts have promoted a model of PSDM. While judges may theoretically arbitrate, evidence from critical care environments suggests that in disputes about children’s best interests they are rarely involved and almost all conflicts are resolved privately. Since PSDM is demanding, and outside pressures may conflict with the best interests principle, empirical studies would be valuable to determine what drives these private processes of PSDM. On a more theoretical level, if we accept that no single decision-making group can lay an indefeasible claim to moral authority, PSDM represents the ‘least bad’ method of making decisions about children. Yet examination of several models of SDM indicates that any positive benefits it offers are to the decision makers, for example, in offering psychological and welfare benefits to parents, and also benefits to society, by promoting social cohesion and a civil society. The child benefits from these only by association with adult beneficiaries, and such benefits may be variable. As the focus of best interests is supposed to be the child, founding a decision-making strategy on the benefits it offers to decision-makers, even if these sometimes percolate to the child, seems incongruent with this. Worse, because children’s voices are absent from the process and their interests are bound to others, PSDM shares the problem with any paediatric decision-making strategy that other interests may be supplant those of the child in the final decision. In hand with the demands PSDM places on decision makers, this incongruence is especially uncomfortable, and it would be reassuring if we could identify positive reasons for PSDM. For instance we may claim that a multiplicity of independent rational arguments with similar conclusions make us more likely to reach the correct answer. Similarly we could suggest that group decision-making reduces bias or that more consequences are imagined. Yet none of these ideas are incontestable and a deeper philosophical investigation should be undertaken. Moreover, the practical process by which PSDM takes place already appear to depend on a participant’s satisfaction of demanding criteria—these may be complicated further by such a philosophical investigation and it is likely that the current process of PSDM will require reform. Perhaps these reasons will not even be practically achievable. Should they not, we may need to radically re-evaluate current processes, we might for instance abandon PSDM as poorly adapted to the paediatric paradigm, or overhaul the best interests test to suggest that children’s interests need not be paramount. Neither of these solutions seems unproblematic. In the meantime, the child’s place in collaborative decision might benefit from allowing a more open recognition that each participant brings competing interests to the decision.

SDM has become orthodoxy in healthcare as a way to make medical decision-making comply with the principle of autonomy. Yet autonomy is a complex issue and SDM cannot simply be grafted onto existing processes without analysis. Analysis now suggests that the benefits of SDM in the care of competent adults may have concurrent risks. PSDM too seems to have become orthodoxy with insufficient consideration of the commitments it involves and the benefits it may accrue to the child. Although it may benefit decision-making parties, much more work needs to be undertaken to determine if these benefits imply unacceptable risks to the interests of the child.

## Footnotes

1 By English law I mean the law in England and Wales. Northern Irish law generally considers children’s best interests in the same way, although there are some differences with Scottish law, especially in its consideration of adolescent consent and refusal.

2 Exactly how much weight a child’s views should be given is a contentious topic. While radical child liberationists argue that children of all ages should possess the same rights as adults, a majority of thinkers hold children’s views are not equal to those of their parents and doctors while the child is immature. Judging when children attain maturity may rely on their ability to understand and process information and formulate reasonable, stable conclusions, abilities of which age is often argued to be an arbitrary measure. These debates shall not be discussed further here, but a good introduction is found in Archard [4] pp. 70–97. For discussion of the somewhat contradictory approach of UK law on this issue see Elliston [25] pp. 47–94.

3 The concept of best interests does not of course have a settled meaning. It is clear that in much of English law best interests are taken to be a person’s preferences (e.g. Mental Capacity Act 2005), although concepts such as suffering are also used in children e.g. [66]. A seminal paper by David DeGrazia explains that best interests can be interpreted in three ways; as personal preferences, as mental states (e.g. pleasure and suffering) or as objective lists (e.g. Rawls’ natural primary goods [60]). None of these theories is without criticism, for instance mental states may be false or transient, preferences may be mistaken and objective lists may be impossible to discover or apply. In the case of those who are unable to communicate we have no access to either their wishes or their mental state, and despite many attempts, there is no generally agreed objective criteria. This epistemological problem of not being able to know what best interests are is thus central to debates about how we should make decisions in PSDM. See DeGrazia [20].

4 An example of this in English law is found in the Children Act 1989s. 1, which states: “the child’s welfare shall be the court’s paramount consideration.” The paramountcy principle has been argued to imply certain unpalatable outcomes, for instance that parents should be doing what is best for a child at the expense of the wider family’s integrity and wellbeing, or paradoxical ones, for instance that we must give every child what is best even when what is available is satisfactory. This paper is not to consider such philosophical conundrums. Instead I shall take it as read that we ought to value children and promote their interests in adult affairs, even if, as some commentators maintain, such promotion means we should allow families independence to make decisions or that we should accept that some children would be better off dead.

5 As a doctrine of common law it is considerably more ancient. The earliest references to a best interests standard lie within child custody law, and the concept of best interests is usually seen as the modern descendant of the sovereign’s *parens patriae* protection. It was in *Blisset’s Case* [1774] the best interest principle was first articulated by Lord Mansfield CJ who stated:” *if the parties disagreed, the court will do what shall appear best for the child”.* When the welfare principle first entered the law in the Guardianship of Infants Act 1925, it was still a principle of custodial adjudication, rather than a general principle in the treatment of children.

6 The nature of this judicial objectivity is widely disputed. For instance see: Baines [5].

7
*Re J [* 66 *] at 41 per Lord Donaldson MR.*

8 The cases referred to in Wyatt are *Re A (Male Sterilisation)* [63] and *Re S (Adult Patient: Sterilisation)* [69] .

9 Therein doctors sought a court order that it would be in severely disabled Charlotte Wyatt’s best interests to withhold ventilation should she require it.

10 A reading of *Glass v UK* also shows attempts at SDM between physicians and parents, suggesting SDM was indeed the norm at this time. Such an approach is also advocated in the near contemporaneous second edition of withdrawal guidelines from the Royal College of Paediatrics and Child Health [72].

11 Another example is *Re T* [70] where a mother successfully argued against her child receiving a liver transplant.

12 Specifically antibiotics, blood sampling and lengthy cardiopulmonary resuscitation.

13 It has been argued that we should concentrate upon the moral justifications of resource scarcity, rather than medical futility and best interests as a means to determine if treatment is continued. A detailed exploration of the implications of such an approach would be worthwhile, however I choose in this paper to concentrate upon the needs of the child rather than the community at large, and thus will not discuss this argument further. See [77, 86].

14 A search of the Westlaw database in June 2013 (using keywords including withdrawal, children, medical, life sustaining treatment, parental consent) produced the following cases where doctors proposed treatment be withdrawn or withheld: *Re K* [67]—a child with a neuromuscular disorder where permission to remove an intravenous line providing nutrition was granted; *Re B* [64]—a child with an unknown metabolic condition where leave to withhold ventilation if required was granted; *Re OT* [68]—a 9 months old child with genetic abnormalities, ventilator dependent, severe brain damage where leave to withdraw ventilation and withhold further treatment was granted; *Re B* [65]—A father who disagreed about withdrawal of ventilation from his son, who had a neurological disorder, the case was resolved when the father withdrew his objection; *NHS Trust v Baby X* [53]—Withdrawal of ventilation from a child with a catastrophic brain injury, although opposed by the family, was allowed; *NHS Trust v H* [54] an advance care plan that limited treatment of a child with a severe brain injury was upheld, despite opposition by his mother. Although not the focus of the search an additional two oncology cases where doctors petitioned to treat against the wishes of the parent were also produced, *An NHS Trust v A* [1] and *An NHS Trust v SR* [3].

15 I do not claim this intransigence is malicious or conniving, and note it may be equally effective when used by parents or clinicians depending on the situation. My main concern is that the child may get lost within the dispute. I will develop this argument as the paper proceeds.

16 I am conducting an empirical project which may begin to answer some of them. ‘Judging Best Interests in the Paediatric Intensive Care Unit: The Location, Basis and Scope of Decision Making (BIPIC)’ is an empirical ethics project supported by a Wellcome Trust Clinical Fellowship in Biomedical Ethics (Grant WT097725MF). It involves qualitative semi-structured interviews with parents of children who have been patients on PICU, members of clinical ethics committees and PICU doctors and nurses to explore their experiences and perceptions of key ethical concepts used in decisions about children in PICU who have no antecedent wishes.

17 While I have focused upon the parents and clinicians, I am grateful to the anonymous reviewer who raised the issue of the extent to which families are in fact involved in the shared decision making process. Roy Gilbar’s recent research [28, 29] highlights the desire of adult decision makers to include significant others in the decision making process, but the reticence of the law and clinicians to recognise the validity of their involvement. While being beyond the scope of this paper, such research is obviously of interest, for instance in conjunction with other explorations of involving extended family in decisions about children. For example Ladd and Mercurio [43] discuss the validity of treatment decisions emanating from the grandparents of neonatal patients, suggesting this may be acceptable since they may offer more mature perspectives. Similar justification may be drawn from Zamvar et al. [88] who note that grandparents may be more physically capable of serving the best interests of infants who are receiving complex therapies in the community.

18 What represents a health need is debatable, for instance, should such need be simply the biological needs of patients or something more holistic that includes their psychological and social needs? Since such a conceptual separation is challenging in children who cannot express a preference or states of mind, in this discussion I interpret health need in the narrowly biological sense, separate from the patient’s motivations and wider psychosocial needs. A clinician may of course be highly alert to these, but their lack of expertise in nonmedical matters is a common criticism of paternalism.

19 To save words I have deliberately not considered Charles et al.’s [15] professional-as-agent model on the grounds that it seems especially weak when applied to infants who have no history or preference on which to base an agent’s decision. This inaccessibility suggests professional-as-agent fails in children on basic epistemological grounds. Professional-as-agent seems strongly allied to models of substituted judgement, which have been extensively criticised even in adults where past preferences are known [31, 33].

20 As we shall see, although SDM may avoid such problems by evoking the patient’s right to autonomy, such problems also persist in PSDM because agreement does not necessarily equal moral legitimacy.

21 For example Ward LJ in *Re A* [62] at 969d.

22 For instance the response to the case of *Re T* [70]. Consider the differing standpoints of Fox and McHale [27], Lyons [45] and Elliston [25] pp. 95–144 about the reasonableness of refusing a therapy for which the risks and benefits are relatively well known.

23 Uncertainty invites the question: ‘how uncertain?’ Rawls’ natural primary goods [60] (on which basic interests are predicated) of health, intelligence and vigour are clearly in need of revision if we are to accommodate disability.

24 Indeed, in English law it assumes this protective purpose in s.31 of the Children Act 1989.

25 It is not the focus of the paper to explore quality of life debates in depth, but we must note the very complex and often contradictory nature of conclusions in the literature, both positive and negative, that are dependent on a multitude of factors. Objective quality of life measures show no concurrence with subjective measures, and arguments that self reported quality of life is inauthentic are inherently controversial. Certainly we must be very cautious whenever quality of life data is presented as it is often subject to considerable spin. Nevertheless, while it is not clear how much the neonatal intensive care studies noted here can tell us about other outcomes—and it is arguable that they are irrelevant to very profoundly disabled children—they nevertheless represent rare longitudinal data. It is also important to note the undermining effect of social inequalities on these figures: Families without effective and adequate support may not do well and positive evidence points to the fact that caregiver and sibling quality of life is best in well supported families.

26 While studies such as EPICure [19] have given us more insight into the health implications for extremely premature infants who survive, we know little directly of the experiences of the families and children of families who insisted on treatment in defiance of medical advice, or allowed non-treatment contrary to their own intuition.

27 An alternative argument is that because the best interests of the child are innately uncertain PSDM reduces both the level and the burden of that uncertainty. The second part of this argument seems to be about unburdening the decision makers, so is a prudential one and remains incongruent with paramountcy. In the first part, since the uncertainty of a decision is either soluble or not, we cannot say that an intrinsically uncertain decision is less uncertain if we share it without offering reasons that sharing reduces this uncertainty. I shall consider such reasons toward the end of this paper.

28 In a review of the definition of futility in adult intensive care, Wilkinson and Savulescu [86] note the difficulty in predicting outcomes when chances of survival chances are in the region of a few percent, chances that may be clinically unacceptable but which appear acceptable to many families. They also observe that, in adult care, even very slim chances of recovery or very great levels of disability may be acceptable to many families.

29 The authors also list a number of extra risks to the patient that SDM entails.

30 I note that Sandman [74] suggests that negotiation poorly describes the process of SDM because SDM is not a bargain between equals where there are gains for both sides but instead is a compromise between the clinician’s altruistic consideration of the patient’s best interests and the patient’s values. While I accept that SDM is a complex interaction, I suggest this critique does not fit a situation where the best interests of the patient are not self evident, while the question of altruism appears obfuscatory in PSDM because each party has an equally tenable claim to be acting altruistically.

31 Such as those concerned with euthanasia and termination of pregnancy.

32 For instance in his chapter on National Ethics Commissions [48] pp. 73–87.

33 I acknowledge some readers may nevertheless find Huxtable’s adoption of the term ‘compromise’ uncomfortable. In their writings on compromise and consensus both Huxtable [38] pp. 127–132 and Moreno [48] pp. 12–13 acknowledge that many philosophers feel these terms have negative connotations when attached to moral validity. I briefly consider the main arguments below.

34 There are other parties we might also consider such as a national or hospital clinical ethics committee. The latter is favoured by Huxtable [38], although whether an institutional body would be viewed as sufficiently independent, or have the degree of authority required requires investigation.

35 More neutrally this could be expressed as a preference for a particular type of society.

36 It is worth noting Richard Miller [46] attempts to use a similar structure of pluralism and liberal theory to accomplish such a justification. Miller roots paediatric medical decision making in the shared guardianship of children’s basic needs (which Miller identifies as physical, intellectual and emotional welfare, respect and rights to an open future), which primarily rest with the parent unless it can be demonstrated a child’s welfare will suffer. While such a theory could support PSDM as an expression of a shared intention of promoting a child’s welfare (although this is a rather anaemic argument), Miller leaves it to rest on the suggestion that provided a family uphold the child’s basic interests, favouring family autonomy is the best way to safeguard children’s welfare, which I have already suggested is not always the case.

37 In other words, a thoroughgoing consequentialist will invest all the moral importance of a decision in its consequences, whereas a similarly committed deontologist will invest none.

38 Of course, working out *which* times will remain tricky, and represents a further problem to be addressed.
